# Qualitative Assessment of Quality and Readability of Patient-Directed Arabic Online Resources for Cochlear Implants in Children

**DOI:** 10.1055/s-0045-1804516

**Published:** 2025-07-01

**Authors:** Saad A. Sanad, Aseel M. Mokhtar, Manar O. Alharbi, Hisham B. Alem, Afnan F. Bukhari, Faisal Zawawi

**Affiliations:** 1 Department of Otolaryngology – Head & Neck Surgery, College of Medicine, King Abdulaziz University, Jeddah, Saudi Arabia; 2 Department of Otolaryngology – Head & Neck Surgery, Prince Sultan Military Medical City (PSMMC), Riyadh, Saudi Arabia; 3 Cochlear Implant Center, King Saud Medical City, Riyadh, Saudi Arabia

**Keywords:** readability, quality, cochlear implant, online information, Arabic language

## Abstract

**Objective** To assess the readability and quality of various websites providing information on cochlear implantation (CI) in children in Arabic.

**Methods** The present is a cross-sectional online search. We conducted searches in the Arab language using the three most popular search engines, Yahoo, Google, and Bing, to search the topics of “cochlear implant” and “cochlear implant in children.” The information quality was evaluated through the DISCERN tool, while readability was examined using the OSMAN readability tool, which incorporated two readability metrics: the automated readability index (ARI) and the Läsbarhetsindex (LIX, reliability index).

**Results** In a study of 50 websites, the median Discerning the Quality of Information on Treatment Choices (DISCERN) quality score was 38, with an interquartile range (IQR) of 29 to 46, suggesting poor quality. Out of the 50 websites evaluated, only 10 were deemed to be of good quality. The median readability ARI was 8th grade (IQR: 6–9), which considered above the level of general population. Certain websites were found to be at or below the suggested 6th grade reading proficiency level. The median LIX was of 69 (IQR: 64–71), indicating that the text is very difficult. There was no correlation between readability and the DISCERN score.

**Conclusions** Our results suggest that many websites offering information on CIs for children in Arabic exhibit varying levels of quality and are composed in language surpassing the general population's reading ability. Developers should focus on improving the readability of their online content to ensure that the average reader can understand and benefit from the information.

## Introduction

Cochlear implants (CIs) stand out from traditional acoustic amplification methods by using electrical stimulation to deliver auditory information to the auditory system. Unlike standard amplification, which relies on enhancing acoustic signals, these implants take a different approach. They bypass the compromised peripheral sensory systems, frequently responsible for hearing impairment, and instead deliver electrical impulses directly to the spiral ganglion. Subsequently, these impulses are relayed to the eighth cranial nerve. 1

Presently, CI stands as the preferred treatment for individuals who are deaf from birth and has become a standard medical procedure for children facing severe-to-profound sensorineural hearing loss. 2 3 4 Across the globe, more than 300 thousand deaf individuals have received CIs. 5 An empirical study from 2003, involving a sample of 10 thousand children in Saudi Arabia under the age of 15 years, showed that 13% of the participants had hearing impairment, and sensorineural hearing loss (SNHL) was found among 142 (1.5%). 6 7

Nowadays, patients and their families have the ability to obtain a wide array of health-related information through the internet, with a substantial number regarding it as their primary source of health information. 8 Approximately 69% of the global population is currently utilizing the internet and, in the Middle East, this figure climbs to 80%. 9 Furthermore, internet utilization has been increasing steadily through the past two decades. 10 In the United States, a substantial 49% of the population has reported conducting online searches for health-related information. 11 Incorrect or deceptive information has the potential to result in individuals overlooking the side effects of medical interventions, postponing the seeking of professional medical assistance for health issues, or opting for unverified treatments instead of established standard therapies. 8

Active patient involvement is essential in contemporary healthcare, ensuring the delivery of comprehensive medical services. 12 For this to happen, precise and ample information concerning health conditions is required. 12 Health literacy is defined as “the degree to which individuals possess the ability to acquire, analyze, and comprehend fundamental health information and services necessary for making suitable health choices.” 13 When communicating written health materials, it's vital to take into account the varying health literacy levels among patients. 14 Typically, it's recommended by the American Medical Association (AMA) and the National Institutes of Health (NIH) that written health materials maintain a reading difficulty level of up to the sixth grade, aligning with the average reading proficiency of adults in the United States. 15 16 17

The Arab world consists of 22 countries, all of which designate Arabic as their main language. Collectively, these nations are home to a population of 600 million individuals. 18 There is a notable scarcity of accessible websites dedicated to the dissemination of trustworthy health-related information within this region. 19 It's worth noting that a significant portion of the Arab world's populace is proficient exclusively in the Arabic language. Consequently, their access to health-related information relies heavily on websites presented in Arabic. 19

The first step in improving the quality of online health information is evaluating it. This study aims to evaluate patient-centered websites in Arabic that cover CIs in children. This will be done by examining the readability and overall quality of information available through popular search engines.

## Methods

The present research constitutes a secondary data analysis, focusing on publicly accessible information. Given the absence of privacy issues, the study received ethical approval exemption from the institutional review board (protocol number 31-22) and informed consent were deemed unnecessary. To identify pertinent websites, the three most utilized search engines were chosen, namely Google, Yahoo, and Bing. The search was executed in November 2022 utilizing the search terms “cochlear implant” and “cochlear implant in children” in the Arabic language as primary search queries. To prevent any potential search bias, location information was deliberately removed, and user accounts were signed out.

For each of the search terms, the initial 50 websites from each search engine were meticulously documented by three distinct authors, resulting in a total of 450 results for both “cochlear implant” and “cochlear implant in children.” The inclusion criteria encompassed websites in Arabic, intended for the public rather than medical professionals, and specifically focused on CI. Conversely, websites meeting the following criteria were excluded: those not discussing CI for children, duplicates, websites primarily featuring videos, inaccessible websites, advertisements, and newspaper articles that did not cover related information. This method is aligned with multiple previous studies. 20 21

**Fig. 1:**
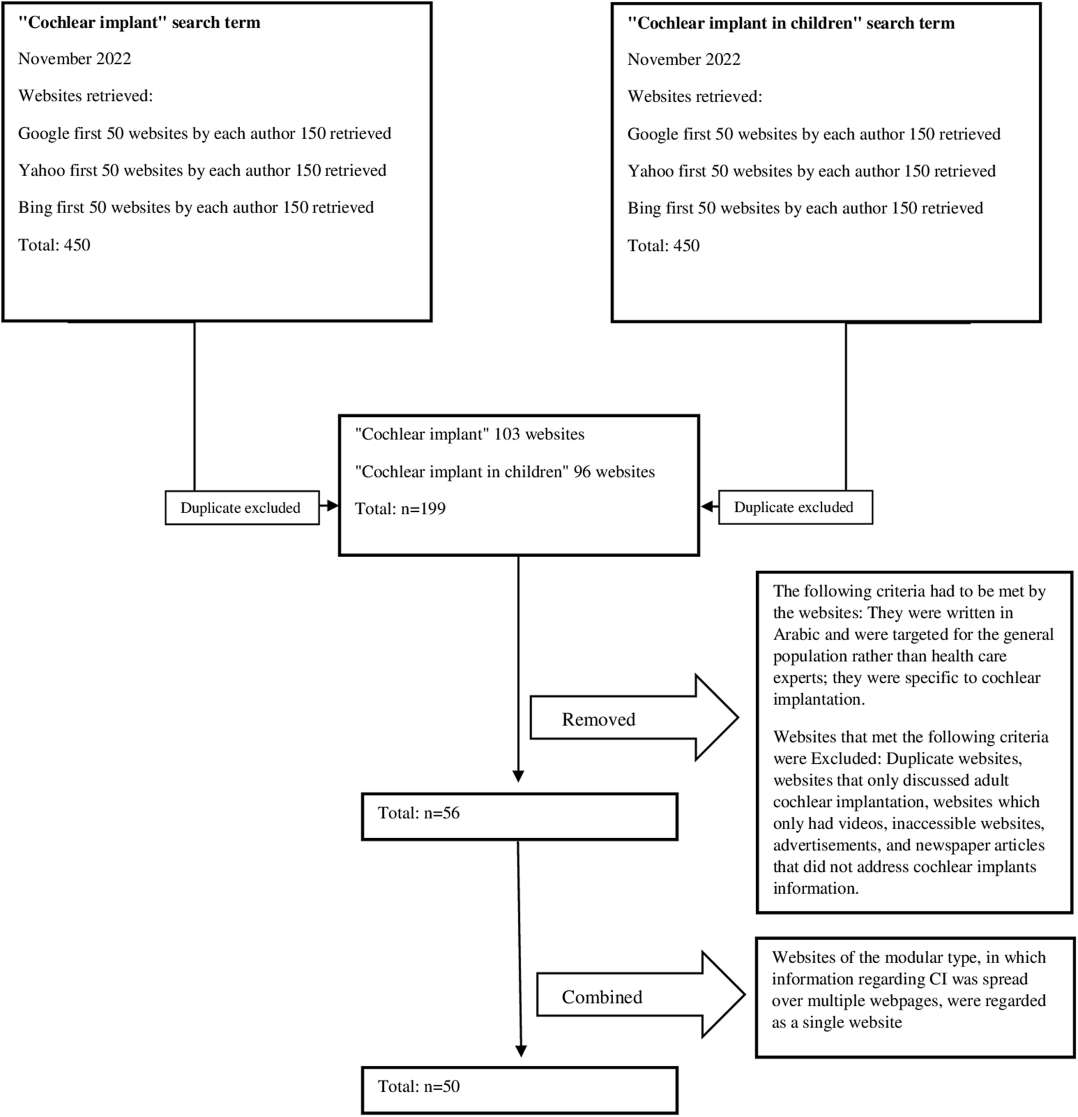
Diagram illustrating the internet search process. The Arabic terms for *cochlear implant* and *cochlear implant in children* were entered into Google, Yahoo, and Bing.

Our search generated 56 websites. Notably, those adopting a modular structure, wherein information regarding CI was dispersed across multiple webpages (such as explaining the risks on one page and the benefits on another), were classified as one website, resulting in 50 websites available for the ultimate analysis ( Fig. 1 ).

## Quality Analysis

We conducted a quality analysis to evaluate the quality of the information available. Recognizing that not all health information available online maintains a high standard, the study incorporated the Discerning the Quality of Information on Treatment Choices (DISCERN) tool, which was developed by the United Kingdom's National Health Service for quality assessment. This tool comprises a validated questionnaire consisting of 15 key questions, including an overarching query regarding the publication's overall quality as an information source. These questions encompass inquiries into publication reliability (1–8) and specific details (9–15). 22

Each question was appraised on a 5-point scale, ranging from “no” (scored as 1) to “yes” (scored as 5). The highest achievable score is 80, with increased scores suggesting more dependable publication. Subsequently, the results were grouped accordingly into different quality tiers, including “excellent” (scores from 68 to 80), “good” (scores from 55 to 67), “fair” (scores from 42 to 54), “poor” (scores from 29 to 41), and “very poor” (scores from 16 to 28), based on the achieved score. 22

To assess the DISCERN score, three independent observers were involved, with any discrepancies resolved before finalization. To assess agreement between raters, the intraclass correlation coefficient (representing absolute agreement) was employed, yielding a value of 0.918, which signifies excellent agreement.

## Readability Analysis

Captions, images, locations, hyperlinks, and advertisements were excluded. For assessing readability, the open-source metric for measuring Arabic narratives (OSMAN, open source), a specific software designed to assess readability for Arabic text, was employed. 23

Furthermore, two readability metrics were utilized. The first is the Läsbarhetsindex (LIX, reliability index), calculated based on the percentage of words longer than six letters plus the average number of words per sentence; the interpretation of the results is presented in Table 1 . 24 The second is the automated readability index (ARI), used for comparing the number of letters within a word to the number of words within a phrase, with results expressed as a school grade level. 24 Both indices are considered language independent and readings are, thus, fairly consistent between the Arabic and English languages. 23

**Table 1:** Läsbarhetsindex (LIX, reliability index)

Score	Meaning
0–24	Very easy
25–43	Easy
35–44	Standard
45–54	Difficult
≥ 55	Very difficult

In contrast to other readability indices, incorporating linguistic factors into the formula results in a decrease in correlation scores. This is evident when examining Flesch scores, specifically noting a diminished correlation between Arabic and English. Applying the Flesch formula to Arabic text will yield inaccurate results due to the syllable count always being zero. This leads to a higher Flesch score, erroneously suggesting the text is easy to read, which is not accurate. 23 A lower score in both indices indicates better readability. 24 The website's word tally was additionally factored in to assess the readability of its content.

## Statistical Analysis

IBM SPSS Statistics for Windows (IBM Corp., Armonk, NY, United States) software, version 25.0, was used for conducting statistical analysis. Descriptive statistics, including median, mean, range, and interquartile range (IQR), were computed as applicable for reporting purposes. A correlation study using Pearson's correlation coefficient was then conducted to explore the relationship between DISCERN and readability scores. Inter-rater reliability (absolute agreement) was assessed using the intraclass correlation coefficient. The threshold for statistical significance was set at *p* < 0.05.

## Results

A total of 50 appropriate websites were found. The websites were analyzed by three separate authors to avoid inadvertent bias. The intraclass correlation coefficient yielded a value of 0.918, signifying excellent agreement.

The median DISCERN score for the entirety of the websites stood at 38, with an IQR from 29 to 46 and a range extending from 20 to 62. Notably, the majority of these websites, totaling 30 (66%), fell within “poor” and “very poor” quality classifications. Conversely, about 20 websites, constituting 34%, achieved scores deemed as “fair” or “good.” Among these, 10 websites were categorized as “fair,” 10 as “good,” and none of the websites received a high enough score to merit the classification of “excellent.” For a detailed list of websites with the highest possible DISCERN ratings, refer to Table 2 .

**Table 2:** Ranking of the top 10 websites based on the DISCERN tool

	DISCERN	ARI	LIX	Word number
https://www.tbeeb.net	62	6.82	65.64	3,342
https://www.audition.guide/ar/cochlear-implant/cochlear-implant-in-algeria/	61	10.64	75.51	2,802
https://esteshary.com	58	11.49	79.46	936
https://enthealtharabia.com	57	6.63	67.49	1,570
https://dailymedicalinfo.com	56	14.4	82.9	1,064
https://www.mayoclinic.org	55	6.37	65.6	1,193
https://sotor.com	54	17.37	93.37	839
https://dailymedicalinfo.com	53	8.67	75.91	933
https://www.audition.guide/ar/cochlear-implant/baby-cochlear-implant/	52	7.68	69.3	1,724
https://amalyat.com	50	6.04	65.24	572

**Abbreviations:** ARI, automated readability index; CI, cochlear implant; LIX, Läsbarhetsindex (reliability index); DISCERN, Discerning the Quality of Information on Treatment Choices.

When considering the 16 questions evaluated by the DISCERN ratings, the highest scores were recorded in questions 9. “Does the website describe how each treatment works?”, 10. “Does the website describe the benefits of each treatment?”, and 11. “Does it describe the risks of each treatment?”. A comprehensive breakdown of scores for all questions can be found in Table 3 .

**Table 3:** Assessment of health information quality on treatment options using the DISCERN criteria

Questions	Median	IQR
1. Are the aims clear?	1	3
2. Does it achieve its aims?	0	5
3. Is it relevant?	3	2
4. is it clear what sources of information were used to compile the publication (other than the author or producer)?	1	1
5. Is it clear when the information used or reported in the publication was produced?	1	2
6. Is it balanced and unbiased?	2	2
7. Does it provide additional sources of support and information?	1	0
8. Does it refer to areas of uncertainty?	2	1
9. Does it describe how each treatment works?	5	0
10. Does it describe the benefits of each treatment?	5	4
11. Does it describe the risks of each treatment?	5	4
12. Does it describe what would happen if no treatment was chosen?	1	0
13. Does it describe how the treatment choices affect overall quality of life?	3	2
14. Is it clear that there may be more than one possible treatment choice?	2	2
15. Does it provide support for shared decision-making?	1	1
16. Based on the answers to all of the above questions, rate the overall quality of the publication as a source of information about treatment choices	3	2
**Total**	38	17

**Abbreviations:** DISCERN, Discerning the Quality of Information on Treatment Choices; IQR, interquartile range.

**Note:** General instructions on the use of the DISCERN instrument are available at http://www.discern.org.uk/general_instructions.php .

**Fig. 2:**
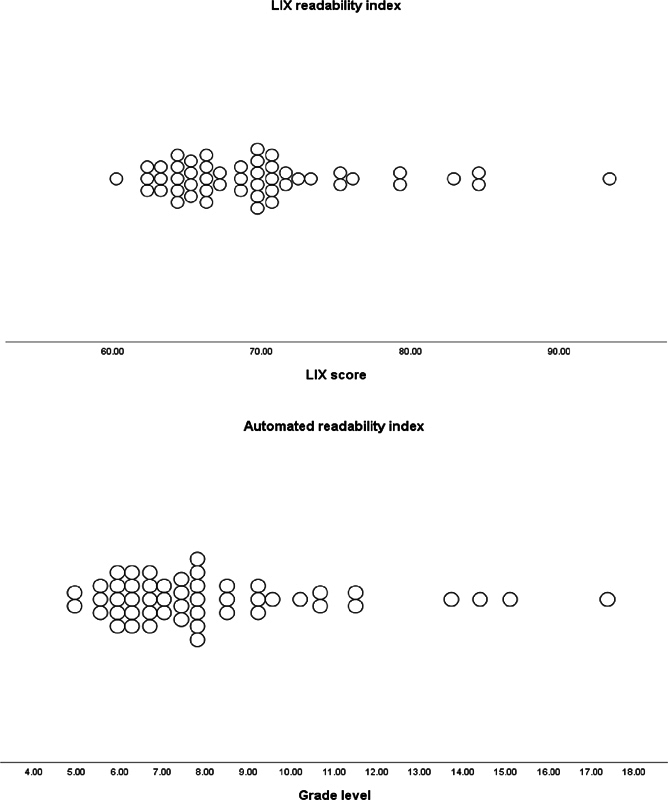
The reading level assessment for 50 websites on cochlear implants in children. Reading levels were assessed 2 validated readability indices (the automated readabilityindex [ARI] and Läsbarhetsindex (LIX, reliability **i** ndex). Below 45 is the recommended LIX, while 6th grade is the recommended level for the ARI.

In terms of readability, the median ARI was 8th grade (IQR: 6–9), surpassing the recommended level by the AMA and NIH. 15 16 The lowest ARI was 5th grade, while the highest was 13th. The median LIX was of 69 (IQR: 64–71), indicating that the text is very difficult to see in Table 1 . The lowest LIX observed was 60, and the highest was of 93. Among the evaluated websites, 21 achieved an ARI score corresponding to a reading level considered adequate for the general population, while none met the standard or easy criteria based on the LIX. The correlation between both readability scores was high (r = 0.947, *p* < 0.05). The total word count ranged from 170 to 3,172, with a median of 906 (IQR: 562–1,699). Refer to Fig. 2 for the distribution of readability scores among the websites.

For websites scoring fair or above according to the DISCERN tool, the median ARI and LIX were 8th grade (IQR: 6–9) and 68 (IQR: 65–78) respectively. In contrast, websites scoring poor or very poor had ARI and LIX of 7th grade (IQR: 6–8) and 68 (IQR: 64–71), respectively. The DISCERN score did not significantly correlate with readability scores, and there was no significant difference among each of the categories ( *p* > 0.05).

## Discussion

Due to the wealth of information, ease of access, and the ability to remain anonymous, increasing numbers of patients are seeking online information about their medical issues. 25 The use of the internet has surged in the last two decades, with over 80% of the United States population now utilizing it. 10 About half of all Americans seek health-related information online. 11 Internet resources for medical information play a significant role in educating people and fostering meaningful discussions with healthcare providers. 8

Despite these advantages, it is crucial to approach internet information with caution. Ensuring that patients consume the highest quality and reliable information, free from technical difficulties and presented in easily understandable language, is essential. 8 The lack of standardization and regulation in available healthcare information online often results in considerable variation in published material. 8

The current investigation reveals that the only Arabic website on CI in children exhibits poor quality and readability, indicating a need for overall improvement.

Our findings revealed that there is a significant variability in quality. The median DISCERN score stood at 38 (IQR: 29–46), suggesting that most of the information assessed was of poor or very poor quality. Such content lacks comprehensiveness exhibits various shortcomings and cannot be regarded as a reliable or appropriate source of health-related information.

While some websites (33.4%) demonstrated moderate quality or better—and have been determined to be valuable sources of information about CIs, albeit with limitations, the overall picture is one of suboptimal quality, this picture is consistent with several previous otolaryngology research studies. 21 26 27

To evaluate the quality of health-related information, DISCERN was chosen. This tool works by assessing factors like the clarity of listed information sources, balance and impartiality, thorough coverage of treatment options with advantages and disadvantages outlined, and encouragement for patients and doctors to make collaborative decisions. A perfect score of 80 would signify an ideal website, but none of the analyzed sites, including those created by respectable organizations and academic institutes, achieved this benchmark. To enhance websites, it is crucial to provide additional information sources, clearly describe treatment options, and outline the risks and benefits associated with every intervention and treatment, as well as that of avoiding therapy, including how they affect the patient's daily activities. Interestingly, our research revealed the benefits and risks of avoiding therapy were only discussed on two of the websites, which adequately covered this aspect.

A considerable difference in the quality of information about CIs for children was observed in our study. Notably, tbeeb.net received the highest score among all 50 websites, with a median of 62, representing 77% of the total possible points. However, while tbeeb.net proved to be more informative than other sites, it still falls short of being the ideal reference for patients. Our study emphasized the substantial differences among websites, underscoring the importance of directing patients to specific sources while cautioning others.

Another crucial criterion for evaluating health-related information found on the internet is how readable the writing is. As mentioned before, the reading level that recommended for written health information is around the 6th grade. 15 16 17 The metrics employed in this study to gauge readability include the automated readability index score and the LIX, both commonly used for assessing the readability of language and online health-related information independently so the results can be fairly compared to other languages. 23 24

In this study, an overwhelming majority of websites, 100% according to the LIX metric, exhibited an average reading grade surpassing the comprehension of the general population. Additionally, all websites had a LIX exceeding 60, categorized as “very difficult.” While this doesn't necessarily prevent individuals with low reading levels from exploring those websites, it does reduce understanding, potentially impacting the patient's expectations and decision-making. When comparing websites of varying quality, no significant difference in readability was observed. This suggests that readability does not necessarily correlate with website quality and vice versa. This observation aligns with findings from several otolaryngology studies. 21 26 28 Medical writers can identify parts that may need to be modified to improve understanding by using readability testing, which determines the written materials' comprehension level.

In an earlier study conducted by the same authors of this study, it was determined that online information regarding CI in children, when presented in the English language, demonstrated substandard quality, and exceeded the comprehension level of the general population. This suggests that the observed issue might be universal rather than specific to a particular region of the world. 21 Even though readability exceeded the recommended level of information in Arabic language as well it is far more readable than English websites. A comprehensive breakdown of the results of both studies is demonstrated in Table 4 .

**Table 4:** A comprehensive breakdown of results of websites for CI in children in both the Arabic and the English languages

	CI for children's websites in Arabic	CI for children's websites in English
DISCERN score	38	33
Highest DISCERN score	62	71
Readability	ARI: 8th grade; LIX: 69	Average of five readability indices; 11th grade
Word count	906	698
Correlation	No correlation between quality and readability	No correlation between quality and readability

**Abbreviations:** ARI, automated readability index; CI, cochlear implant; LIX, Läsbarhetsindex (reliability index); DISCERN, Discerning the Quality of Information on Treatment Choices.

This study is subject to certain inherent limitations. The choice of keywords used to select websites may differ from the terms employed by patients, potentially impacting the inclusiveness of the search. Important elements, including the readers' motivation, curiosity, and background knowledge, or the visual elements and layout of the page, were not taken into consideration by the readability test used here. Additionally, the literacy levels of the Arabic-speaking population have not been extensively studied to serve as a reference point.

Furthermore, despite being verified for evaluating website quality, the DISCERN tool has numerous limitations. Because scoring depends on the subjectivity of the observer, bias may be present. To mitigate this, the websites were assessed by three independent observers with a high interrater reliability. However, the DISCERN tool does not consider how information is presented or the ease of navigation on a specific website. Another limitation is the absence of a survey analyzing parents' understanding of CIs in children based on the material presented on each website. Future studies should involve patients, comparing how they search for information about this topic online, then assessing the knowledge gained after reading each website.

## Conclusion

In the present era, the internet serves as a valuable tool for acquiring information on diseases, as well as their prevention and treatment. It is imperative that such information is sourced from reliable, easily accessible, and trustworthy platforms. To our knowledge, this study represents the first attempt to assess the quality and readability of online resources related to CIs for children in Arabic. The overall findings indicate that the material presented on these websites is challenging to comprehend and surpasses the recommended reading level for health information. Furthermore, most of the evaluated websites lacked sufficient information to assist patients in making informed medical decisions.

Considering the increasing number of parents utilizing the internet to gather medical information for their children, the necessity for high-quality, easily understandable websites in their native language cannot be overstated. Immediate measures are required to address websites offering Arabic health information on CIs for children. Ongoing efforts by medical societies are essential to enhance the quality and readability of online information for patients. The recommendations derived from this study can be utilized by these websites to improve their content. Health professionals play a crucial role in endorsing and supporting the development of websites that are not only easy to read but also provide high-quality information.
